# The psychometric properties of the Vietnamese Version of the Five Facet Mindfulness Questionnaire

**DOI:** 10.1186/s40359-022-01003-3

**Published:** 2022-12-12

**Authors:** Hang T. M. Nguyen, Hoang V. Nguyen, Thai T. H. Bui

**Affiliations:** 1 Faculty of Psychology, VNU University of Social Sciences and Humanities, Hanoi, Vietnam; 2 Department of Psychology, University of Minnesota, Minneapolis, USA

**Keywords:** FFMQ, Mindfulness, Psychometric properties, Confirmatory factor analysis

## Abstract

**Background:**

Although recent decades have witnessed a growing interest in mindfulness with the development of many mindfulness scales and their adaptation to different cultures, there has been no attempt at developing or adapting a mindfulness scale for Vietnamese people. To fill this gap and encourage the study of mindfulness in Vietnam, we adapted a 20-item short-form of the Five Facet Mindfulness Questionnaire (FFMQ-20) into Vietnamese, which we called the FFMQ-V, and examined its psychometric properties in a series of three independent studies.

**Methods:**

In Study 1, using a college sample (*N* = 412) we conducted several exploratory factor analyses to elucidate the factor structure of the FFMQ-V. In Study 2, using an independent college sample (*N* = 344) we performed a confirmatory factor analysis (CFA) to test the goodness-of-fit for all obtained factor models from Study 1. In this study, we also examined the discriminant validities of the FFMQ-V by correlating mindfulness and other related psychological constructs, including acceptance, nonattachment, depression, anxiety, and stress. In Study 3, we replicated all data analyses in Study 2 using a community sample of young adults (*N* = 574).

**Results:**

Across all Studies, our results indicated that the hierarchical five-factor model with method factors best captured the latent structure of the FFMQ-V. Our results also showed that the mindfulness facets met our expectations as they correlated positively with the acceptance and nonattachment and negatively with the depression, anxiety, and stress.

**Conclusions:**

In aggregate, our EFA and CFA results provided strong evidence for the hierarchical five-factor model with method factors in both community and college samples, suggesting that the FFMQ-V can be used to measure trait mindfulness of the Vietnamese young adults.

**Supplementary Information:**

The online version contains supplementary material available at 10.1186/s40359-022-01003-3.

## Background

For the past few decades, mindfulness research has enjoyed a rapid growth in interest and publication [1]. Several review studies [2, 3] concluded that trait mindfulness was negatively associated with many health issues such as chronic pain, depression, social anxiety, and emotion regulation and that mindfulness training could bring about positive psychological effects. As promoted by the growing evidence and consensus on their effectiveness, mindfulness-based therapies have been applied more widely both in clinical practice [4] and in non-clinical settings that aimed to improve physical and mental health of the general population [5–13]. These wide applications of mindfulness in turn have advocated for a more refined understanding and a better measurement of this construct, resulting in varied interpretations of mindfulness and many mindfulness scales [14] such as the Five Facet Mindfulness Questionnaire (FFMQ) [15, 16], the Freiburg Mindfulness Inventory (FMI) [17], and the Mindful Attention Awareness Scale (MAAS) [18]. Among these measurements, the FFMQ is perhaps the most widely used instrument as it has been adapted to many cultures (see [19, 20] for a review). Specifically, the FFMQ conceptualizes mindfulness as a psychological construct with five latent facets: Observe (i.e., attending to internal and external experiences), Describe (i.e., labeling internal experiences with words), Actaware (i.e., attending to one’s activities of the moment), Nonjudge (i.e., taking a nonevaluative stance toward thoughts and feelings), and Nonreact (i.e., not getting caught up in thoughts and feelings). Although the FFMQ offered a psychologically interpretable model of mindfulness, many researchers [19–21] disagreed on the latent structure of the FFMQ in the general population and several authors [19–22] also called attention to the lack of the FFMQ research in non-Western countries. In the following paragraphs, we examined each of these points in details.

More than a decade ago, Baer et al. [16] introduced the FFMQ that was constructed by pooling the best items in terms of factor loadings from the five preexisting mindfulness measurements [17, 18, 23–25] using factor analysis techniques [26, 27]. Interestingly, Baer et al. [16] showed that although the FFMQ had a clear five-facet structure with an overarching mindfulness construct in a sample of meditators, when administered to non-meditator samples, a hierarchical four-factor model (i.e., without the Observe facet) was a better fit for the FFMQ. Unfortunately, the latent structure of the FFMQ remains a contested topic as Lecuona et al. [19] reported that out of 36 studies on the psychometric properties of the FFMQ that they found (i.e., excluding the original paper by Baer et al. [16]), 25 confirmed the original hierarchical five-factor FFMQ model, 8 suggested alterative structures, and 3 proposed person-centered analyses. For instance, several authors [28, 29] showed that a non-hierarchical five-factor model was a better alternative to the hierarchical five-factor model, whereas Aguado et al. [30] suggested that their bifactor model of mindfulness outperformed the non-hierarchical five-factor model (see also [31]). To investigate these and several other alternative FFMQ models, Lecuona et al. [19] conducted a conceptual replication study [32] that found a strong support for the bifactor model of mindfulness with (1) four facets (i.e., without the Observe facet) and (2) two method factors, which were latent factors that accounted for variance due to methodological instead of theoretical effects.

To add more nuances to the current discussion on the FFMQ structure, a recent study by Lecuona et al. [21] that used a recently developed psychometric method (i.e., the exploratory graph analysis (EGA) [33–35]) made several contributions to the understanding of the FFMQ structure. Specifically, using the EGA, which was shown to be comparable to standard parallel analysis [35], Lecuona et al. [21] offered a six-facet model with the Actaware split into two facets, which was deemed a competitive alternative to the regular five-facet model. These results corresponded to Karl et al. [20]’s findings that in many cultures, the six-factor model with a split Actaware facet provided the best fit model for the FFMQ. These and other diverged results on the latent structure of the FFMQ led several authors [20, 22, 36, 37] to reexamine the contemporary approach to mindfulness assessment (including the FFMQ) that was mostly based on research conducted in Western countries.

As mentioned earlier, Karl et al. [20] conducted a cross-cultural study to investigate the FFMQ latent structure across 16 countries with a total sample of 8541 participants. Among them, only 1051 (approximately 12%) participants were recruited from non-Western countries (i.e., China and India). Interestingly, Karl et al. [20] noted that although the hierarchical five-factor FFMQ model with uncorrelated method factors showed good fit in most cultures, such a model showed below acceptable fit in a number of cultures, mostly non-Western, which suggested that “the ideal structure of the FFMQ might be driven by cultural values” (p. 1233). Furthermore, Karl et al. [20]’s observation on the underrepresentation of non-Western cultures, especially Eastern cultures, in the FFMQ research highly corresponded to the results reported by Lecuona et al. [19]. Specifically, out of 37 studies (i.e., Baer et al. [16] and 36 follow-up FFMQ studies), only three studies recruited their participants from non-Western countries (i.e., Bhutan, China, and Japan) with 2219 participants (approximately 13% of the pooled sample from all studies). To put these results in a broader context of trait mindfulness research, Karl and Fischer [22] reported that “the publications on trait mindfulness were substantially biased towards Europe, Australia, and North America” (p. 1364). For instance, among the five most productive countries, Australia, Canada, the United Kingdom, and the United States of America (USA) together produced 60.72% of all published documents on trait mindfulness, whereas China accounted for 10.17% only. Other non-Western countries in Africa and Asia accounted for a small portion of all published documents.

Although Western countries did not claim the monopoly of mindfulness research, virtually all countries derived their conceptualization of mindfulness from Western psychology’s present-centered interpretations that reduced mindfulness to bare-attention and nonjudging [18, 37–39]. For instance, Karl and Fischer [22] showed that in both China and the USA (i.e., the two countries that produced the highest number of publications on mindfulness), most frequently cited articles on mindfulness were those by Baer et al. [16], Bishop et al. [38], Brown and Ryan [18], and Kabat-Zinn [39]. Among these highly influential and homogenous group of authors, Brown and Ryan [18] defined mindfulness as “the state of being attentive to and aware of what is taking place in the present” (p. 822) and published the MAAS, which was later used by Baer et al. [15, 16] to construct the FFMQ. However, supported by diverged evidence on the latent structure of the FFMQ and their interpretation of Buddhist teachings, few theorists [37, 40] argued against the conceptualization of mindfulness as “an instrumental tool: heightened, value neutral form of concentrated attention” [40, p. 8]. Rather than considering mindfulness as a psychological trait, Purser and Milillo [37] encouraged researchers to elevate mindfulness to “a form of ethics-based mind training” (p. 7). Although such a call to reconceptualizing Western understanding of mindfulness and thus changing the present approach to mindfulness assessment (including the FFMQ) will require more time to gain popularity, it is undoubtful that the understanding of mindfulness as a psychological construct will benefit greatly from encouraging mindfulness research in many non-Western countries, the first step of which should be adapting and evaluating the FFMQ in such countries.

### Research on mindfulness in Vietnam

Although many Vietnamese meditation schools were developed in the early history and mindfulness has been practiced by both Buddhists and non-Buddhists, Vietnamese mindfulness research has only began in recent decade and produced few studies [41–45]. However, most of these studies used strictly qualitative research methods and did not include any well-established mindfulness scale. For instance, although Nguyen [45] used the FMI to investigate the influence of mindfulness on life satisfaction and nonattachment, the author did not examine the validity of the FMI using psychometric tools such as confirmatory factor analysis (CFA, [27]). Unfortunately, to our best knowledge, there has been no attempt at developing or adapting a mindfulness scale for Vietnamese people, which limits the study of mindfulness in the context of Vietnamese culture to qualitative studies only. Thus, we believe that it is necessary to adapt a well-established measurement of mindfulness with known psychometric properties into Vietnamese to encourage better research on mindfulness in Vietnam.

Due to the theoretical comprehensiveness of the FFMQ, its known psychometric properties, and its popular usage, we choose to adapt the FFMQ into Vietnamese. More specifically, we propose to adapt the 20-item short-form of the FFMQ (FFMQ-20, [46]) as the FFMQ-20 possesses good psychometric properties in both college and community samples (see also [47, 48]). To adapt the FFMQ-20, we conducted a series of three studies in which we translated the FFMQ-20 into Vietnamese and examined its reliability, content validity, discriminant validity, and construct validity using both college and community samples.

The remainder of the present paper is organized as follows. In the “Methods” section we first describe a series of three studies. In Study 1, we translated the FFMQ-20 into Vietnamese (i.e., we refer to the (Vietnamese) translated FFMQ-20 as the FFMQ-V) and examined the latent structure of the FFMQ-V using exploratory factor analysis (EFA, [26]) in a college sample (*N* = 412). Next, to test the factor structure of the FFMQ-V in Study 2 we conducted a CFA using an independent college sample (*N* = 344). In this study, we also examined the discriminant validities of the FFMQ-V. We then describe our Study 3, in which we replicated Study 2 using an independent community sample (*N* = 574). Finally, we summarize our results for the three studies before discussing the latent structure of the FFMQ-V and its validation.

## Methods

### Samples and procedures

In Study 1, we began our adaptation of the FFMQ-20 by recruiting 412 college students (85% female, 15% male), aged 20-to-26 years (*M* = 20.94, *SD* = 1.02). Among these students, approximately 47% were freshmen, 30% sophomores, 20% juniors, and 3% seniors. Most participants came from rural areas (62%), many from urban areas (26%), and few from mountainous areas (12%). All participants were informed about the goals of the study, and that their voluntary participation allowed them to withdraw from the study at any point should they wish to do so. Furthermore, no confidential information was obtained to ensure that all participants remained anonymous. After giving their consent, each participant was asked to complete the FFMQ-V.

In Study 2, we investigated the construct validity of the FFMQ-V using the CFA in an independent sample of 344 college students (87.5% female, 12.5% male), aged 20-to-24 years (*M* = 20.99, *SD* = 1.02). Among these students, 49.4% were freshmen, 24.1% sophomores, 24.4% juniors, and 2.0% seniors. Furthermore, 58.7% of participants came from the rural area, 29.4% from the urban area, and 11.9% from the mountainous area. Like Study 1, participation was voluntary and anonymous. After giving their consent, each participant was asked to complete all questionnaires that are described below and additional demographic items.

In Study 3, we investigated the construct validity of the FFMQ-V using the CFA in an independent community sample of 574 young adults (54.7% female, 44.4% male, and 0.9% others), aged 18-to-25 years (M = 20.55, SD = 2.33). Among these participants, approximately 2.8% finished no more than secondary school, 48.8% received high school education, 43.7% held a university diploma, and 4.7% completed higher education beyond the university diploma. Furthermore, approximately 49.1% came from rural areas, 44.9% grew up in urban areas, and 5.9% lived in mountainous areas. Moreover, approximately 53.5% were students, 21.4% were labor workers, and 25.1% were intellectuals (e.g., teachers, medical doctors, and businessmen). Most participants (93.9%) indicated that they did not have an affiliate with any religion. To recruit our participants, we distributed our questionnaires to schools, a university, hospitals, factories, business offices, government administration offices, and an army unit. Like previous studies, participation was voluntary and anonymous. After giving their consent, each participant was asked to complete all questionnaires described below and additional demographic items.

### Measures

The Five Facet Mindfulness Questionnaires by Tran et al. [46] included five latent factors: (1) Observe, (2) Describe, (3) Actaware, (4) Nonjudge, and (5) Nonreact. Each factor is identified by four items in a 5-point Likert scale (1 = *never or very rarely true*, 5 = *very often or always true*). Example items include “*I pay attention to sensations, such as the wind in my hair or sun on my face*” (Observe), “*My natural tendency is to put my experiences into words*” (Describe), “*I don’t pay attention to what I’m doing because I’m daydreaming, worrying, or otherwise distracted*” (Actaware; recorded), “*I believe some of my thoughts are abnormal or bad and I shouldn’t think that way*” (Nonjudge; recorded), and “*When I have distressing thoughts or images, I feel calm soon after*” (Nonreact; recorded). Following Tran et al. [46], we recoded all inverse items before computing total scores.

To translate the FFMQ-20 into Vietnamese, we adopted the backward translation design [49] in which we first recruited two Vietnamese psychologists to translate the FFMQ-20 into Vietnamese and obtained a provisional FFMQ-V. Next, we asked two independent researchers to back-translate the provisional FFMQ-V into English. We then compared the original FFMQ-20 with the back-translated FFMQ-V to examine the suitability of the source language version of the test. Finally, we administered the FFMQ-V to five Vietnamese college students to survey their opinions on (1) the use of language (i.e., were items clear and comprehensible?) and (2) cultural appropriateness (i.e., did items imply any cultural offense to responders?). Based on responses from these students, we replaced few didactic words with more commonly used terms and obtained our final version of the FFMQ-V (see Table 1).

**Table 1:** The Vietnamese Five-Facet Mindfulness Questionnaire

Subscale	FFMQ-20 Item	Vietnamese adaptation
Actaware	1*	Khi làm việc gì đó, tâm trí tôi như đi lang thang làm tôi dễ bị phân tâm, không thể tập trung
	2*	Tôi không chú ý vào việc mình đang làm vì tôi lơ mơ (mơ ngày), lo lắng hoặc bị phân tâm
	4*	Tôi dễ bị mất tập trung
	8*	Tôi rất khó tập trung vào những gì hiện đang xảy ra
Observe	6	Tôi chú ý vào cảm giác cơ thể và cảm nhận được gió thổi vào tóc hay ánh nắng chiếu vào mặt
	15	Tôi chú ý đến các âm thanh như tiếng kim đồng hồ chạy, tiếng chim kêu hay tiếng xe cộ
	10	Tôi để ý đến mùi và hương của mọi vật xung quanh
	17	Tôi nhận ra các hình ảnh (thị giác) trong nghệ thuật và trong tự nhiên, chẳng hạn như màu sắc, hình dáng, kết cấu, các sắc thái của ánh sáng và bóng tối
Nonjudge	16*	Tôi nghĩ rằng, một số cảm xúc của tôi không phù hợp và tôi không nên cảm thấy như vậy
	14*	Tôi tự nhủ: mình không nên nghĩ như mình đang nghĩ
	5*	Tôi tin rằng, một số suy nghĩ của tôi không bình thường hoặc xấu và tôi không nên nghĩ như vậy
	19*	Khi trong đầu tôi xuất hiện các hình ảnh gây đau buồn, khi đó tôi thường đánh giá bản thân tốt hay xấu phụ thuộc vào tính chất của các hình ảnh đó
Describe	7*	Tôi rất khó tìm từ để mô tả chính xác tôi đang cảm thấy như thế nào
	12*	Khi có một cảm giác nào đó trên cơ thể, tôi rất khó mô tả nó vì tôi không tìm được từ ngữ thích hợp
	20	Nhìn chung, tôi có thể mô tả cảm giác hiện tại của mình một cách chi tiết
	18	Thiên hướng tự nhiên của tôi là lấy kinh nghiệm của bản thân đưa vào lời nói của mình
Nonreact	9	Khi trong đầu tôi xuất hiện các ý nghĩ hoặc hình ảnh gây đau buồn, tôi dừng lại và nhận thức rõ về ý nghĩ hay hình ảnh đó mà không bị chúng kéo đi
	11	Trong những tình huống khó khăn hay căng thẳng, tôi có thể dừng lại mà không phản ứng ngay lập tức
	13	Khi trong đầu tôi xuất hiện các ý nghĩ hoặc hình ảnh đau buồn, tôi nhanh chóng lấy lại được cảm giác bình an
	3	Tôi quan sát cảm xúc của mình mà không bị cuốn theo chúng

FFMQ-20 denotes the 20-item short-form version of the Five-Facet Mindfulness Questionnaire and * denotes recoded item

The Acceptance and Action Questionnaire (AAQ-II, [50]) consists of seven items and measures acceptance, experiential avoidance, and psychological flexibility. Each item was scored from 1 (*never true*) to 6 (*always true*). Example items include *“My painful experiences and memories make it difficult for me to live a life that I would value”* and *“Worries get in the way of my success.”* In our studies, we used the Vietnamese adapted AAQ-II by Nguyễn et al. [51].

The Nonattachment Scale is a 7-item scale (NAS, [52]) that measures nonattachment, a Buddhist concept that describe as “a flexible, balanced way of relating to one’s experiences without clinging to or suppressing them” (p. 820). Each item was scored from 1 (strongly disagree) to 6 (strongly agree). Example items include “*I can enjoy pleasant experiences without needing them to last forever*,” “*I can enjoy my family and friends without feeling I need to hang on to them*” and “*I do not get “hung up” on wanting an “ideal” or “perfect” life.”* In our studies, we used the Vietnamese adapted NAS by Nguyen and Nguyen [53].

The Depression, Anxiety, and Stress Scale (DASS, [54]) consists of three subscales that measure the core psychological symptoms of depression, anxiety and stress. Each subscale contains seven items, each of which is scored from 0 (did not apply to me at all) to 3 (applied to me very much or most of the time). Example items include “*I felt that I had nothing to look forward to*” (Depression), “*I was worried about situations in which I might panic and make a fool of myself*” (Anxiety), and “*I was intolerant of anything that kept me from getting on with what I was doing*” (Stress). In our studies, we used the Vietnamese adapted DASS by Nguyễn et al. [51].

Furthermore, based on the theoretical relations between mindfulness and psychological constructs such as acceptance, nonattachment, and mental health issues [2, 28, 29, 52, 55, 56], we hypothesized that the FFMQ-V correlated positively with the AAQ-II and the NAS and negatively with the DASS subscales. For each scale used, we reported its reliability in the “Results” section.

### Missing data and data analyses

All three studies had relatively low rates of missing data (see Table 2). For each study sample, we imputed missing data using the predictive mean matching (PMM) method [57, 58] as simulation results showed that the PMM method worked well across a wide range of missing data situations [59].

**Table 2:** Missing data rates for three Vietnamese Five-Facet Mindfulness Questionnaire Studies

Study	# of Items	No missing	Missing = 1	Missing > 1	Max missing
1 (N = 412)	24	340 (82.5%)	45 (10.9%)	27 (6.6%)	6
2 (N = 344)	59	270 (78.5%)	48 (14.0%)	26 (7.5%)	5
3 (N = 574)	61	518 (90.2%)	44 (7.7%)	12 (2.1%)	10

^#^ of Items denotes the total number of items that was administered. No Missing denotes the number of participants who completed all items. Missing = 1 denotes the number of participants who omitted only one item. Missing > 1 denotes the number of participants who omitted more than one item. Max Missing denotes the maximum number of items that participants missed

Moving on with our Study 1, we first computed the Kaiser–Meyer–Olkin’s (KMO, [60]) measure of sampling adequacy and conducted the Bartlett 's test of sphericity [61] to examine the suitability of our sample for EFA. Next, we determined the optimal number of factors to extract by performing a parallel analysis [62]. Based on these results, we performed an EFA using polychoric correlations (i.e., we included the correlation matrix in Additional file 1: Table S1 of the online supplement) and the unweighted least square estimator. Next, we rotated the extracted factor loadings using the oblimin algorithm from 1,000 random starts [63]. Furthermore, following our literature review of the FFMQ we also investigated the hierarchical structure of the FFMQ-V by conducting an exploratory bifactor analysis using the Schmid-Leiman transformation [64]. Finally, for each factor in each model we investigated (1) its reliability by computing Cronbach’s α, @@@Guttman’s λ_6_, and/or McDonald’s ω (see [65] for a review) and/or (2) its factor score indeterminacy (see [66] for a review). All analyses were conducted in R programming language [67] with the fungible [68], mice [69], and psych [70] packages.

Based on Study 1 results, in Study 2 we investigated the latent structure of the FFMQ-V by conducting a series of confirmatory factor analyses, in which we fitted three CFA models: (1) a five-factor model, (2) a hierarchical five-factor model, and (3) a hierarchical five-factor model with two method factors (i.e., one factor for positively worded items and another for negatively worded items). All CFA models were estimated using the weighted least square mean and variance adjusted with data treated as ordinal. Furthermore, for each fitted CFA model we computed a number of fit indices: (1) the Chi-squared test for goodness of fit, (2) the Comparative Fit Index (CFI, [71]), (3) the Tucker-Lewis Index (TLI, [72]), (4) the Root Mean Squared Error of Approximation (*RMSEA*, [73]), and (5) the Standardized Root Mean Square Residual (*SRMR*; [74]). Following Hu and Bentler [73] we adopted cutoff values of .90 for the *TLI* and the *CFI*, cutoff values of .08 for the *RMSEA*, and cutoff values of .08 for the *SRMR*.

Furthermore, to investigate the discriminant validity of the FFMQ-V, we computed the Pearson correlations between the FFMQ-V and other measurements of related psychological constructs such as acceptance, nonattachment, depression, anxiety, and stress. Next, we investigated how well each mindfulness facet could predict each of the related constructs using univariate linear regression analyses. The reliability of all scales was also examined using Cronbach’s α, Guttman’s λ_6_, and McDonald’s ω. All analyses were performed in R with supporting packages, including fungible [68], lavaan [75], mice [69], and psych [70].

Finally, in Study 3 we replicated all data analyses performed in Study 2 using a community sample. Note that the online supplement includes the correlation matrices for Studies 2 and 3 in Additional file 1: Tables S3 and S5, respectively.

## Results

### Study 1: Exploratory study using college sample

Our results suggested that an EFA was suitable to our data set (i.e., the KMO value for our sample was 0.73 and a Bartlett’s test of our sample resulted in *χ2* = 1746.02, *df* = 190, *p* < 0.001). Furthermore, our parallel analysis results suggested five common factors (see Additional file 1: Figure S1 of the online supplement). Below, we report only our EFA solution in Table 3.

**Table 3:** The Oblimin Factor Solution of the Vietnamese Five-Facet Mindfulness Questionnaire in College Sample (*N* = 412)

	F1	F2	F3	F4	F5
Items	Actaware	Observe	Nonjudge	Describe	Nonreact
	*Oblimin solution*				
1*	**0.87**	0.01	− 0.04	− 0.04	− 0.01
2*	**0.75**	− 0.01	0.01	0.05	0.09
4*	**0.68**	0.00	0.03	0.01	− 0.06
8*	**0.54**	0.07	0.25	0.04	− 0.05
6	0.02	**0.72**	− 0.12	0.06	0.05
15	0.00	**0.61**	0.09	− 0.12	− 0.11
10	0.08	**0.56**	− 0.13	− 0.03	0.13
17	− 0.03	**0.44**	0.23	− 0.07	0.07
7*	0.10	− .014	**0.72**	0.05	0.03
12*	0.08	− 0.02	**0.63**	0.06	− 0.10
20	− 0.10	0.23	**0.49**	0.03	0.18
18	− 0.06	0.27	**0.31**	− 0.21	0.12
16*	0.00	− 0.05	− 0.02	**0.79**	0.04
14*	− 0.04	0.10	0.08	**0.65**	− 0.10
5*	0.14	− 0.02	0.05	**0.48**	0.04
19*	0.09	− 0.06	0.11	**0.30**	− 0.01
9	0.07	0.03	− 0.03	− 0.02	**0.58**
11	− 0.07	0.03	− 0.02	− 0.03	**0.52**
13	0.02	− 0.06	0.12	− 0.01	**0.44**
3	− 0.07	0.11	0.12	− 0.01	**0.31**
α	0.95	0.88	0.87	0.50	0.70
λ_6_	0.94	0.86	0.86	0.68	0.68
FSI	0.92	0.87	0.87	0.88	0.79
	*Factor correlation coefficients*				
F1	1.00	− 0.02	0.35	0.28	0.00
F2		1.00	− 0.01	− 0.31	0.37
F3			1.00	0.23	0.06
F4				1.00	− 0.18

*Denotes recoded item, α denotes Cronbach’s alpha and λ_6_ denotes Guttman’s λ_6_. FSI denotes the Guttman’s factor score indeterminacy, and N denotes sample size. Factor loadings larger than or equal to 0.30 are in bold font

Scanning Table 3, we observed a clear five-factor structure of the FFMQ-V in the college sample of Study 1. Specifically, the Actaware, Observe, and Nonjudge factors were identified mostly by moderate-to-strong indicators, whereas the Describe and Nonreact factors were identified by weak-to-medium indicators. Interestingly, the salient and cross loadings of item 18 on the Observe, Describe, and Nonjudge factors were relatively small and equal, suggesting that this item might not identify the Describe factor solely. Furthermore, the salient factor loading of item 19 slightly fell below the commonly used cutoff of 0.30 (i.e., it was rounded up to 0.30), suggesting that it was not a good indicator of the Nonjudge factor.

Regarding the reliability of the FFMQ-V, Cronbach’s α and Guttman’s λ_6_ values showed that the Acaware, Observe, Nonjudge, and Describe subscales had moderate internal consistency, whereas the Nonreact subscale had the lowest internal consistency. Turning to the factor score indeterminacy of the FFMQ-V factors, recall that the Guttman’s FSI measures how well estimated factor scores (i.e., sum scores are factor scores) can predict the “true” factor scores such that lower FSI values imply that factor score estimates do not estimate the “true” factor scores well. Our results showed that the Nonreact factor had relatively low FSI, suggesting that its items were not optimal in measuring the Nonreact facet of the FFMQ-V.

**Fig. 1:**
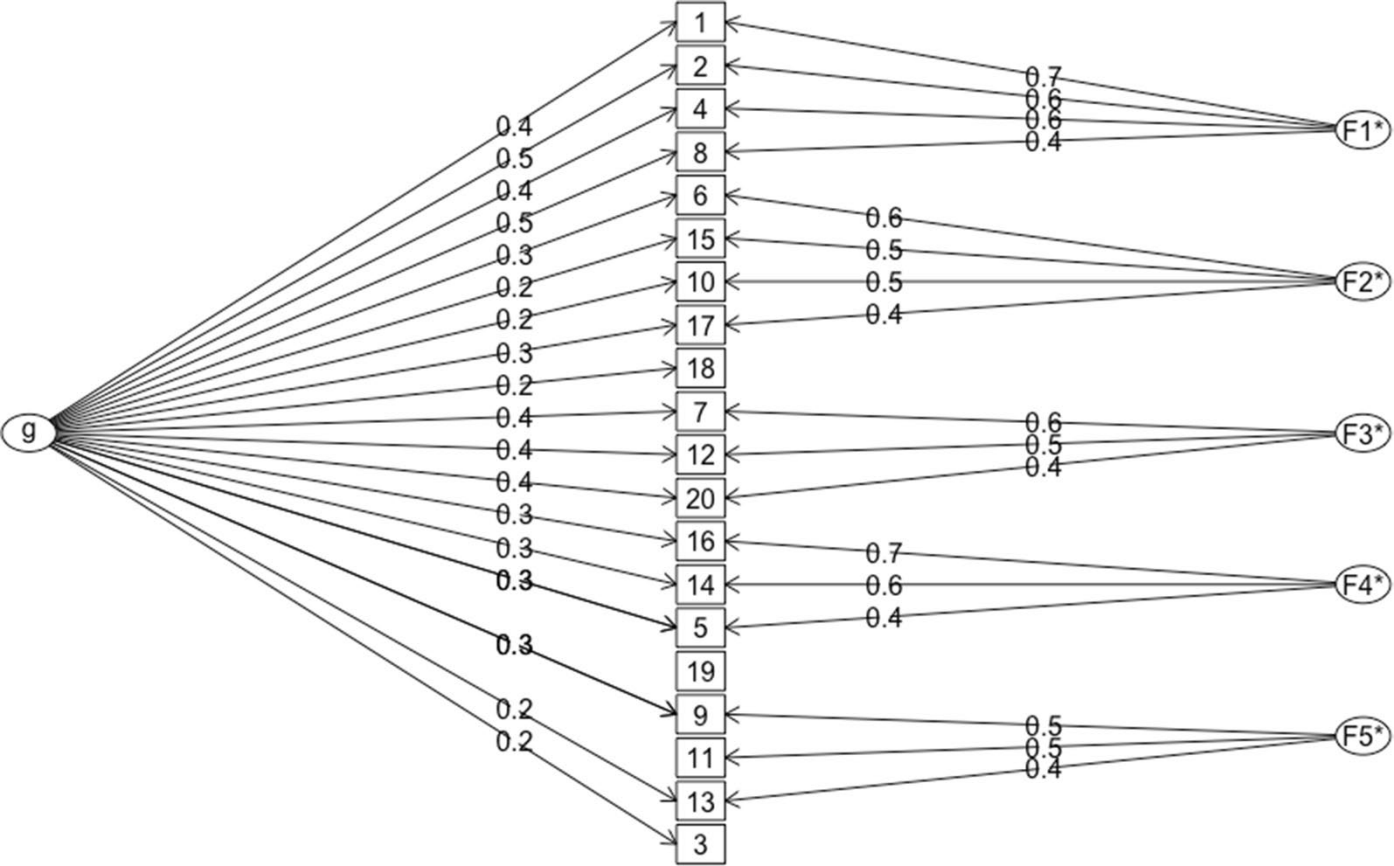
The Bifactor Model of the Vietnamese Five-Factor Mindfulness Questionnaire in College Sample (*N* = 412). *Note* g denotes the general factor, F1* through F5* denote the Actaware, Observe, Nonjudge, Describe, and Nonreact group factors, respectively, and *N* denotes sample size. McDonald’s ω total for total scores and subscales were 0.80, 0.81, 0.62, 0.66, 0.58, and 0.50, respectively

Moving on, Table 3 showed that not all intercorrelations among the FFMQ-V factors were zeroes, which suggested the existence of a higher factor structure of the FFMQ-V. To investigate this possibility, we conducted an exploratory bifactor analysis of the FFMQ-V and reported its results in Fig. 1.

Scanning Fig. 1, we observed a clear latent structure of the FFMQ-V with one general and five group factors. All group factors were identified by at least three items and their reliability coefficients (McDonald’s ω) ranged from 0.50 to 0.80. Although the Nonreact factor had low reliability, the general FFMQ-V was reliable. Finally, similar to the non-hierarchical five-factor results, items 18 and 19 had relatively small factor loadings (i.e., their group factor loadings were less than 0.30). An ad-hoc translational review revealed that the syntax and expressions of both items 18 and 19 required a lengthy un-Vietnamized sentences to fully convey their original meanings. Although our student reviewers had suggested that we could clarify the wording of these items by providing examples, we decided to remain faithful to the FFMQ-20, which might have resulted in confusing expression and thus weak factor loadings of items 18 and 19. Due to these findings, we decided to exclude items 18 and 19 from further analyses.

### Study 2: Confirmatory study using college sample

We summarize our CFA results in Table 4.

**Table 4:** Confirmatory Factor Models of the Vietnamese Five-Facet Mindfulness Questionnaire in College Sample (*N* = 344)

Models	*χ*^2^, *df*, *p*	*CFI*	*TLI*	*RMSEA (*90% *CI), pclose*	*SRMR*
5F	355.77, 125, < 0.001	0.91	0.89	0.07 (0.06, 0.08), < 0.001	0.07
H-5F	389.70, 130, < 0.001	0.90	0.88	0.08 (0.07, 0.09), < 0.001	0.08
H-5FM	158.63, 109, 0.001	0.98	0.97	0.04 (0.02, 0.05), 0.97	0.04

Models 5F, H-5F, and H-5FM denote the Five-factor, the Hierarchical Five-factor, and the Hierarchical Five-factor with method factors models, respectively. *χ*^2^ denotes the chi-square test value for the goodness of fit; *df* denotes the degree of freedom; *p* denotes the *p*-value of the chi-squared test; *TLI* denotes the Tucker-Lewis index; *CFI* denotes the comparative fit index; *RMSEA* denotes the root mean square error of approximation; CI denotes confidence interval; *pclose* denotes the probability that the value *RMSEA* is smaller than or equal to .05; *SRMR* denotes the standardized root mean square residual. NA denotes not applicable due to uncoverged model estimation

**Fig. 2:**
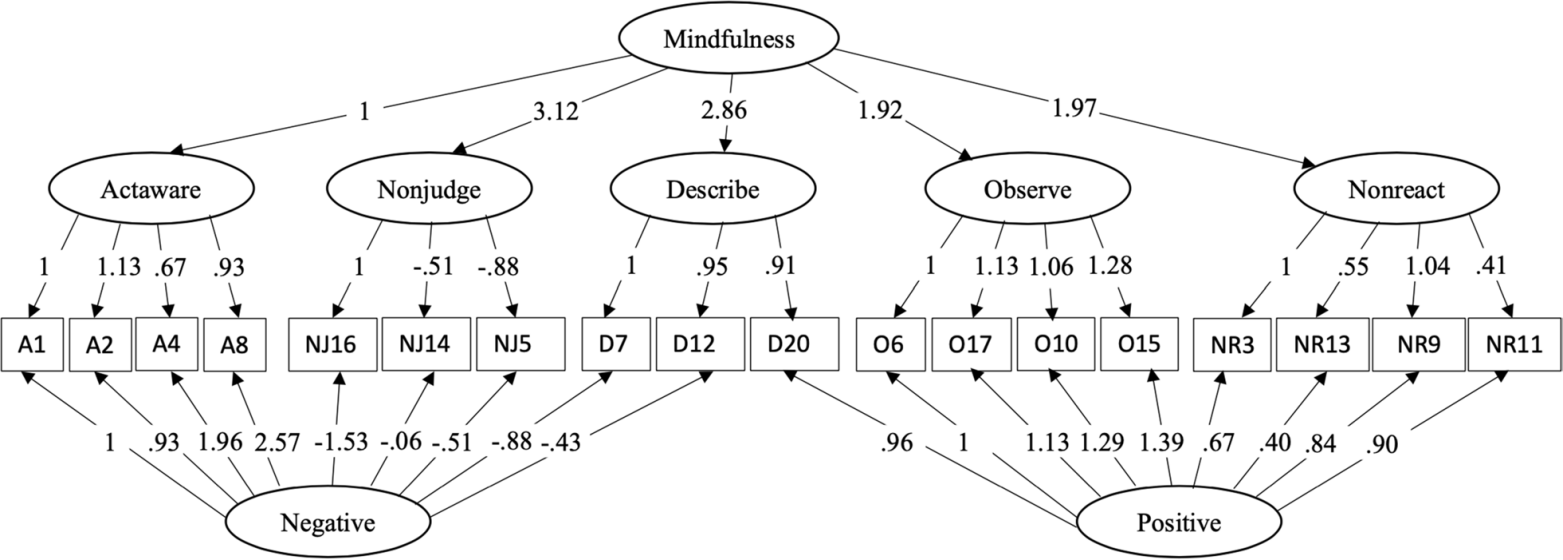
The Hierarchical Five-Factor Confirmatory Factor Model with Method Factors of the FFMQ-V in College Sample (*N* = 344). *Note* The general factor is Mindfulness and four group factors include Actaware (A), Nonjudge (NJ), Describe (D), Observe (O), and Nonreact (NR)

Across all fitted models, the hierarchical five-factor model with method factors (H-5FM) stood out as its χ^2^ test for goodness-of-fit was nonsignificant at the significance level of 0.001 (i.e., this model fitted within sampling error) and all fit indices of the H-5FM indicated excellent fit. We summarize the factor loadings of the H-5FM in Fig. 2.

Regarding the discriminant validity of the FFMQ-V, Table 5 summarizes the intercorrelations among the five FFMQ-V subscales and their intercorrelations with scales of related constructs, including acceptance (AAQ-II), nonattachment (NAS), and mental health issues (DASS).

**Table 5:** Correlation coefficients among mindfulness facets and related constructs in college sample (*N* = 344)

	FFMQ-V								DASS			
	Total	Actaware	Observe	Nonjudge	Describe	Nonreact	AAQ	NAS	Total	Depression	Anxiety	Stress
Total		0.66*	0.44*	0.56*	0.60*	0.46*	− 0.53*	0.35*	− 0.48*	− 0.46*	− 0.43*	− 0.42*
Actaware			− 0.03	0.35*	0.35*	0.04	− 0 .44*	0.16*	− 0.43*	− 0.42*	− 0.38*	− 0.39*
Observe				− 0.10	− 0.01	0.25*	− 0 .01	0.20*	0.04	0.00	0.05	0.06
Nonjudge					0.31*	0.00	− 0 .50*	0.11	− 0.46*	− 0.39*	− 0.46*	− 0.42*
Describe						0.05	− 0 .37*	0.20*	− 0.33*	− 0.34*	− 0.30*	− 0.27*
Nonreact							− 0 .14***	0.30*	− 0.12***	− 0.11	− 0.10	− 0.14***
*Reliability coefficients*												
α	0.71	0.81	0.68	0.71	0.65	0.48	0.86	0.83	0.93	0.84	0.79	0.86
λ_6_	0.77	0.77	0.62	0.62	0.58	0.43	0.86	0.82	0.94	0.83	0.78	0.85
ω	0.82	0.85	0.72	0.75	0.73	0.57	0.88	0.85	0.95	0.88	0.80	0.86

FFMQ-V denotes the Vietnamese Five-Facet Mindfulness Questionnaire, AAQ denotes the Acceptance and Action Questionnaire, NAS denotes the Nonattachment Scale, and DASS denotes the Depression, Anxiety, and Stress Scale. Total denotes a total sum score. α denotes Cronbach’s alpha, λ_6_ denotes Guttman’s λ_6_, ω denotes McDonald’s ω, and *N* denotes sample size. * denotes *p* < .001, ** denotes *p* < .01, and *** *p* < .05

Our results show that the FFMQ-V correlated moderately with all other scales. Specifically, the Actaware, Nonjudge, and Describe subscales had weak-to-moderate correlations with the AAQ-II and the DASS subscales such that higher scores in the Actaware, Nonjudge, and Describe dimensions were associated with (1) higher acceptance scores (i.e., all AAQ-II items were negatively keyed) and (2) lower depression, anxiety, and stress scores. In contrast, the Observe and Nonreact subscales had weak-to-zero correlations with the AAQ-II and the DASS subscales. Finally, all FFMQ-V subscales correlated weakly with the nonattachment scale (i.e., correlation coefficients ranged from approximately 0.10 to 0.30).

Moving on, Table 6 summarizes our regression analyses that independently regressed acceptance, nonattachment, depression, anxiety, and stress on each of the FFMQ-V subscale.

**Table 6:** Linear regression models of predicting acceptance, nonattachment, and mental health using mindfulness facets in college sample (*N* = 344)

				DASS		
FFMQ-V	Estimates	AAQ	NAS	Depression	Anxiety	Stress
Total	B (SE)	− 0.52* (0.04)	0.29* (0.04)	− 0.33* (0.03)	− 0.29* (0.03)	− 0.25* (0.03)
	R^2^	0.28	0.12	0.21	0.18	0.18
Actaware	B (SE)	− 1.03* (0.11)	0.31* (0.11)	− 0.72* (0.08)	− 0.62* (0.08)	− 0.56* (0.07)
	R^2^	0.19	0.02	0.18	0.15	0.15
Observe	B (SE)	− 0.02 (0.13)	0.39* (0.11)	0.00 (0.09)	0.09 (.09)	0.09 (0.08)
	R^2^	0.00	0.04	0.00	0.00	0.00
Nonjudge	B (SE)	− 1.38* (0.13)	0.25*** (0.13)	− 0.79* (0.10)	− 0.87* (0.09)	− 0.72* (0.08)
	R^2^	0.25	0.01	0.15	0.21	0.18
Describe	B (SE)	− 1.10* (0.15)	0.50* (0.14)	− 0.75* (0.11)	− 0.63* (0.11)	− 0.50* (0.10)
	R^2^	0.14	0.04	0.11	0.09	0.07
Nonreact	B (SE)	− 0.43** (0.16)	0.78* (0.13)	− 0.24*** (0.12)	− 0.20 (0.11)	− 0.26** (0.10)
	R^2^	0.02	0.09	0.01	0.01	0.02

FFMQ denotes the Vietnamese Five-Facet Mindfulness Questionnaire; NAS denotes the Nonattachment Scale, AAQ denotes the Acceptance and Action Questionnaire; DASS denotes the Depression, Anxiety, and Stress Scale, and Total denotes the sum score of the FFMQ-V. *N* denotes sample size, B denotes regression coefficient, SE denotes standard error, and *R*^*2*^ denotes the proportion of variation that was accounted for by the model. * denotes *p* < .001, ** denotes *p* < .01, and *** *p* < .05

Our results showed that the FFMQ-V sum score could predict approximately 30% variation of acceptance as measured by the AAQ-II. Among the FFMQ-V subscales, the Actaware, Nonjudge, and Describe scores could independently account for a small proportion of variance in the AAQ-II scores (i.e., the R^2^ ranged from 0.14 to 0.25) and in DASS scores (i.e., the *R*^2^ ranged from 0.07 to 0.21). Surprisingly, these FFMQ-V scores (i.e., the Actaware, Nonjudge, and Describe scores) could independently account for small percentage (i.e., less then 5%) of the variance of nonattachment score. Finally, the Observe and Nonreact scores hardly accounted for the variance of any psychological constructs included in the present study.

### Study 3: Confirmatory study using community sample

As mentioned earlier, in Study 3 we refitted all Study 2 CFA models using an independent community sample and we summarize these results in Table 7.

**Table 7:** Confirmatory Factor Models of the Vietnamese Five-Facet Mindfulness Questionnaire in Community Sample (*N* = 574)

Models	*χ*^2^, *df*, *p*	*CFI*	*TLI*	*RMSEA (*90% *CI), pclose*	*SRMR*
5F	989.64, 125, < 0.001	0.78	0.73	0.11 (0.10, 0.12), < 0.001	0.08
H-5F	1259.03, 130, < 0.001	0.71	0.66	0.12 (0.12, 0.13), < 0.001	0.10
H-5FM	229.04, 109, < 0.001	0.97	0.96	0.04 (0.04, 0.05), < 0.90	0.03

Models 5F, H-5F, and H-5FM denote the Five-factor, the Hierarchical Five-factor, and the Hierarchical Five-factor with method factors models, respectively. χ^2^ denotes the chi-square test value for the goodness of fit; df denotes the degree of freedom; *p* denotes the *p*-value of the chi-squared test; TLI denotes the Tucker-Lewis index; CFI denotes the comparative fit index; RMSEA denotes the root mean square error of approximation; CI denotes confidence interval; pclose denotes the probability that the value RMSEA is smaller than or equal to .05; SRMR denotes the standardized root mean square residual

Our results showed that although the five-factor (5F) and the hierarchical five-factor (H-5F) models did not fit our data, the H-5FM model fit well (i.e., CFI = .97, TLI = .96, RMSEA = .04, and SRMR = .03). These findings conformed to Study 2 results, which showed that both 5F and H-5F models barely fitted the college sample, whereas the H-5FM model fitted well. Moving on to the discriminant validity of the FFMQ-V (see Table 8), our results showed that in general a higher trait mindfulness level was associated with (a) a higher acceptance level (i.e., recall that all AAQ-II items were negatively keyed), (b) a higher nonattachment level, and (c) lower levels of depression, anxiety, and stress. Note that although the Observe subscale correlated positively with the AQQ-II (i.e., those who had higher Observe score tended to be less accepting), such a correlation was small. However, similar to Study 2 results, both Observe and Nonreact facets correlated very weakly with the DASS subscales. Interestingly, Table 8 also showed that the Observe, Nonjudge, and Describe subscales were less reliable in the community sample than in the college sample as their ω values ranged from .48 to .57.

**Table 8:** Correlation coefficients among mindfulness facets and related constructs in community sample (*N* = 574)

	FFMQ-V								DASS			
	Total	Actaware	Observe	Nonjudge	Describe	Nonreact	AAQ	NAS	Total	Depression	Anxiety	Stress
Total		0.64*	0.39*	0.39*	0.66*	0.46*	− 0.49*	0.33*	− 0.46*	− 0.49*	− 0.41*	− 0.42*
Actaware			− 0.20*	0.41*	0.51*	− 0.06	− 0.56*	0.14*	− 0.53*	− 0.54*	− 0.48*	− 0.50*
Observe				− 0.31*	− 0.06	0.39*	0.17*	0.22*	0.12*	0.06	0.12**	0.17*
Nonjudge					0.32*	− 0.27*	− 0.39*	− 0.14*	− 0.33*	− 0.31*	− 0.30*	− 0.34*
Describe						0.03	− 0.50*	0.20*	− 0.41*	− 0.40*	− 0.37*	− 0.41*
Nonreact							− 0.05	0.37*	− 0.08	− 0.10**	− 0.07	− 0.06
*Reliability coefficients*												
α	0.79	0.80	0.66	0.59	0.53	0.53	0.89	0.82	0.92	0.92	0.93	0.97
λ_6_	0.82	0.76	0.60	0.49	0.46	0.47	0.88	0.81	0.91	0.91	0.93	0.97
ω	0.78	0.71	0.57	0.66	0.48	0.53	0.91	0.85	0.98	0.94	0.96	0.91

FFMQ-V denotes the Vietnamese Five-Facet Mindfulness Questionnaire, AAQ denotes the Acceptance and Action Questionnaire, NAS denotes the Nonattachment Scale, and DASS denotes the Depression, Anxiety, and Stress Scale. Total denotes a total sum score. α denotes Cronbach’s alpha, λ_6_ denotes Guttman’s λ_6_, ω denotes McDonald’s ω, and *N* denotes sample size. * denotes *p* < .001, ** denotes *p* < .01, and *** *p* < .05

Next, we summarize our regression results in Table 9. Overall, the FFMQ-V total score could account for 24% of variance in the AAQ-II score, which was similar to Study 2 results (i.e., in Study 2, the FFMQ-V total score accounted for 28% of the AAQ-II score). Unsurprisingly, our regression models also showed that trait mindfulness could not explain nonattachment well as the FFMQ-V total score only accounted for approximately 14% of the variance of the NAS score. Regarding the relationship between mindfulness and mental health, our results showed that the Observe and Nonreact subscales hardly accounted for any variance of the DASS subscales (i.e., the R^2^ for these models were around zero), whereas the Actaware and Describe subscales accounted for more variance of the DASS subscales in community sample than in college sample. Finally, compared to the college sample, the Nonjudge subscale accounted for less variance of the Depression (i.e., R^2^ = .10), the Anxiety (i.e., R^2^ = .09), and the Stress (i.e., R^2^ = .12).

**Table 9:** Linear regression models of predicting acceptance, nonattachment, and mental health using mindfulness facets in community sample (*N* = 574)

				DASS		
FFMQ-V	Estimates	AAQ	NAS	Depression	Anxiety	Stress
Total	B (SE)	− 0.53* (0.04)	0.28* (0.03)	− 0.51* (0.04)	− 0.40* (0.04)	− 0.42* (0.04)
	R^2^	0.24	0.11	0.24	0.17	0.18
Actaware	B (SE)	− 1.36* (0.08)	0.27* (0.08)	− 1.26* (0.08)	− 1.05* (0.08)	− 1.13* (0.08)
	R^2^	0.32	0.02	0.29	0.23	0.25
Observe	B (SE)	0.41 (0.10)	0.42* (0.08)	0.15 (0.01)	0.25 (0.09)	0.37 (0.09)
	R^2^	0.03	0.05	0.00	0.01	0.03
Nonjudge	B (SE)	− 1.20* (0.12)	− 0.34* (0.10)	− 0.93* (0.12)	− 0.84* (0.11)	− 0.99* (0.11)
	R^2^	0.15	0.02	0.10	0.09	0.12
Describe	B (SE)	− 1.16* (0.12)	0.52* (0.11)	− 1.23* (0.12)	− 1.06* (0.11)	− 1.21* (0.11)
	R^2^	0.25	0.04	0.16	0.13	0.16
Nonreact	B (SE)	− .14 (0.11)	0.81* (0.08)	− 0.27*** (0.11)	− 0.17 (0.10)	− 0.15 (0.11)
	R^2^	0.00	0.14	0.01	0.00	0.00

*FFMQ* denotes the Vietnamese Five-Facet Mindfulness Questionnaire; *NAS* denotes the Nonattachment Scale, AAQ denotes the Acceptance and Action Questionnaire; *DASS* denotes the Depression, Anxiety, and Stress Scale, and Total denotes the sum score of the FFMQ-V. N denotes sample size, *B* denotes regression coefficient, SE denotes standard error, and R^2^ denotes the proportion of variation that was accounted for by the model. * denotes *p* < .001, ** denotes *p* < .01, and *** *p* < .05

## Discussion

Using one community and two college samples with a total of 1330 young adults, we conducted a series of studies that adapted the 20-item short-form of the Five-Facet Mindfulness Questionnaire [46] to Vietnamese. Overall, our EFA and CFA results provided strong evidence for the hierarchical five-factor model with method factors in both community and college samples, suggesting that the FFMQ-V can be used to measure trait mindfulness of the Vietnamese young adults. Interestingly, our results suggested excluding items 18 (Describe) and 19 (Nonjudge) from the FFMQ-V as our translation of these items was ambiguous for many participants. However, these results were anything but unique as previous validation studies of the FFMQ-20 also provided diverged suggestions on the number of items to retain. For instance, in their Chinese adaptation of the FFMQ-20 Meng et al. [76] retained all 20 items, whereas Cheong et al. [77] kept only 15 items in their Korean validation. Interestingly, when validating the FFMQ-20 in American adolescents, Abujaradeh et al. [78] suggested removing the entire Observe subscale (with four items) and one Describe item. Once again, these results lend support to Karl et al.’s [20] opinion that the structure and thus measurement of the FFMQ may be culturally oriented. However, none of the aforementioned authors included factor models with method factors in their studies. Thus, such differences regarding the retained number of the FFMQ-20 items may possibly highlight the need for further cross-cultural investigation of the latent structure of the FFMQ-20, as well as the FFMQ, that also examined the role of method factors.

Among the five facets of the FFMQ-V, our results showed that the Nonreact facet had low internal consistency in both college and community samples. These results were unsurprising as two out of the four items in the Nonreact facet (i.e., items 3 and 11) were originally designed to use with experienced meditators (i.e., these items correspond to items 25 and 26 of the FMI, respectively), which lend support for Tran et al.’s [46] suggestion of revising the Nonreact facet. Furthermore, our results showed that the Observe, Nonjudge, and Describe subscales were less reliable in the community sample than in the college sample. Such declining results on the reliability could stem from the diversity in educational backgrounds (i.e., more than half of all participants did not attend college) and in occupational trainings (i.e., approximately a fifth of all participants were labor workers) of the community sample.

When computing the intercorrelations among the five facets of the FFMQ-V, we noted that in the college sample the Observe factor did not correlate with other factors, whereas in the community sample, it correlated moderately with the Actaware, Nonjudge, and Nonreact factors. Commenting on this phenomenon, Baer et al. [15] and other researchers [28, 79] noted that although the Observe factor is a component of the mindfulness definition, it only correlated with the other FFMQ factors when measuring mindfulness of experienced practitioners. In addition to these results, Gu et al. [80] opined that researchers should consider excluding the Observing subscale from comparisons of total scale/subscale scores before and after treatment by Mindfulness Based Cognitive Therapy in clinical samples. When combined with these results, our findings provided further evidence that the Observe facet may not stable across many samples. Nonetheless, the FFMQ-V met our expectations as it correlated positively with the AAQ-II (i.e., the acceptance scale) and negatively with the DASS (i.e., the depression, anxiety, and stress subscales). Although the Nonreact facet had the strongest correlation with the NAS among the five facets of the FFMQ-V, its correlation was weak, approximating only 0.40. In aggregate, these results conform to previous findings [15, 16, 28, 29, 56, 81–84] and suggest that the FFMQ-V had good discriminant validities and can be used to evaluate trait mindfulness in both community and college samples.

Like all other studies, our validation of the FFMQ-20 shared several limitations. First, although the male-to-female ratio in the community sample was approximately one, most student participants in Studies 1 and 2 were female (i.e., approximately 80% student participants were females). To promptly address this limitation, for each FFMQ-V item we computed the average score difference by gender (see Additional file 1: Tables S2, S4, and S6 in the online supplement) and found that on average the magnitude of the average score difference between male and female was 0.14 for Study 1 and 0.37 for Study 2 (on a scale of 5). Thus, for replication studies of the FFMQ-V we recommend that future researchers balance the male-to-female ratio in their samples. A second limitation of our studies was that our studies adapted a short-form version of the FFMQ and thus we suggest that future researchers consider adapting the original FFMQ by Baer et al. [16] into Vietnamese.

## Conclusions

The present study offered a Vietnamese adaptation of the FFMQ-20 that retained 18 items with five subscales. Our version showed strong psychometric properties and thus can be used to measure trait mindfulness of the Vietnamese young adults. Additionally, we recommend that future researchers adapt the original FFMQ into Vietnamese using larger community samples.

## Supplementary Information

**Additional file 1**. The Psychometric Properties of the Vietnamese Version of the Five Facet Mindfulness Questionnaire: Online Supplement.

## Data Availability

The datasets used and/or analyzed during the current study are available from the corresponding author on reasonable request.

## Abbreviations

5F: Five-factor Model
AAQ-II: Acceptance and Action Questionnaire
CFA: Confirmatory Factor Analysis
CFI: Comparative Fit Index
DASS: Depression, Anxiety, and Stress Scale
EFA: Exploratory Factor Analysis
EGA: Exploratory Graph Analysis
FFMQ: Five Facet Mindfulness Questionnaire
FFMQ-20: 20-Item Five Facet Mindfulness Questionnaire
FFMQ-V: Vietnamese Adaptation of FFMQ-20
FMI: Freiburg Mindfulness Inventory
FSI: Guttman’s Factor Score Indeterminacy
H-5F: Hierarchical Five-factor Model
H5-FM: Hierarchical Five-factor Model with Method Factors
KMO: Kaiser–Meyer–Olkin’s Measure of Sampling Adequacy
NAS: Nonattachment Scale
PMM: Predictive Mean Matching
RMSEA: Root Mean Squared Error of Approximation
SRMR: Standardized Root Mean Square Residual
TLI: Tucker–Lewis Index
