# Supplementary material for: The psychometric properties of the Vietnamese Version of the Five Facet Mindfulness Questionnaire
Source: BMC Psychol. 2022 Dec 12;10:300. doi: 10.1186/s40359-022-01003-3 (PMC9743563; doi:10.1186/s40359-022-01003-3)
Supplement: Supplementary file 1 — Additional file 1. The Psychometric Properties of the Vietnamese Version of the Five Facet Mindfulness Questionnaire: Online Supplement. [file 40359_2022_1003_MOESM1_ESM.pdf]

The Psychometric Properties of the Vietnamese Version of the Five Facet Mindfulness Questionnaire

Online Supplement

Hang T.M. Nguyen* Hoang V. Nguyen† Thai T.H. Bui‡

29 September 2022

Contents

Study 1: An Exploratory Factor Analysis in College Sample **4**
The Correlation Matrix of the FFMQ-V 4
Parallel Analysis of the FFMQ-V 6
Score Differences of the FFMQ-V by Gender and Birth Place 7

Study 2: A Confirmatory Factor Analysis in College Sample **8**
The Correlation Matrix of the FFMQ-V 8
Score Differences of the FFMQ-V by Gender and Birth Place 10

Study 3: A Confirmatory Factor Analysis in Community Sample **11**
The Correlation Matrix of the FFMQ-V 11
Score Differences of the FFMQ-V by Gender, Birth Place, and Occupation 13

*VNU University of Social Sciences and Humanities, Vietnam, ntmhang@vnu.edu.vn
†University of Minnesota, USA, nguy2338@umn.edu
‡VNU University of Social Sciences and Humanities, Vietnam, thaibth@vnu.edu.vn

List of Figures

S1 Parallel Analysis of the FFMQ-V 6

List of Tables

S1 Pearson Correlations among FFMQ-V items in College Sample (N = 412) . 5
S2 Average Score Differences by Gender and Birth Place of the FFMQ-V items 7
S3 Pearson Correlations among FFMQ-V items in College Sample (N = 344) . 9
S4 Average Score Differences by Gender and Birth Place of the FFMQ-V items in College Sample (N = 344) 10
S5 Pearson Correlations among FFMQ-V items in Community Sample (N = 574) 12
S6 Average Score Differences by Gender, Birth Place, and Occupation of the FFMQ-V items in Community Sample (N = 574) 14

Study 1: An Exploratory Factor Analysis in College Sample

In this section, we report additional Study 1 results that we performed in our analyses of the Vietnamese Five-Facet Mindfulness Questionnaire (FFMQ-V).

The Correlation Matrix of the FFMQ-V

Table S1 reports the polychoric correlations among FFMQ-V items.

Table S1: Pearson Correlations among FFMQ-V items in College Sample (N = 412)

	A1	A2	A3	A4	A5	A6	A7	A8	A9	A10	A11	A12	A13	A14	A15	A16	A17	A18	A19	A20
A1	1.00	0.69	-0.06	0.55	0.23	0.02	0.29	0.50	0.07	-0.03	-0.10	0.19	0.05	0.11	0.01	0.16	0.01	0.01	0.12	0.08
A2	0.69	1.00	0.02	0.52	0.25	-0.03	0.27	0.45	0.05	0.03	0.01	0.27	0.07	0.17	-0.05	0.19	0.05	0.04	0.15	0.10
A3	-0.06	0.02	1.00	0.01	-0.02	0.12	0.07	-0.01	0.13	0.20	0.20	-0.08	0.22	-0.07	0.11	-0.10	0.20	0.17	0.09	0.19
A4	0.55	0.52	0.01	1.00	0.24	-0.05	0.25	0.49	-0.01	0.03	-0.10	0.24	0.01	0.12	-0.05	0.15	0.00	-0.07	0.14	0.08
A5	0.23	0.25	-0.02	0.24	1.00	-0.06	0.23	0.23	-0.03	-0.19	-0.07	0.16	-0.02	0.34	-0.15	0.41	-0.06	-0.15	0.19	0.13
A6	0.02	-0.03	0.12	-0.05	-0.06	1.00	-0.21	-0.03	0.20	0.44	0.20	-0.10	0.10	-0.13	0.45	-0.20	0.33	0.18	-0.05	0.20
A7	0.29	0.27	0.07	0.25	0.23	-0.21	1.00	0.41	0.03	-0.17	-0.04	0.53	0.09	0.22	-0.01	0.19	0.07	0.09	0.28	0.31
A8	0.50	0.45	-0.01	0.49	0.23	-0.03	0.41	1.00	0.03	0.04	-0.03	0.39	0.08	0.13	0.05	0.20	0.10	0.08	0.26	0.05
A9	0.07	0.05	0.13	-0.01	-0.03	0.20	0.03	0.03	1.00	0.27	0.33	-0.01	0.33	-0.09	0.11	-0.08	0.14	0.20	-0.11	0.10
A10	-0.03	0.03	0.20	0.03	-0.19	0.44	-0.17	0.04	0.27	1.00	0.26	-0.10	-0.01	-0.07	0.38	-0.21	0.24	0.20	-0.14	0.13
A11	-0.10	0.01	0.20	-0.10	-0.07	0.20	-0.04	-0.03	0.33	0.26	1.00	-0.08	0.15	-0.10	0.00	-0.15	0.12	0.16	-0.07	0.18
A12	0.19	0.27	-0.08	0.24	0.16	-0.10	0.53	0.39	-0.01	-0.10	-0.08	1.00	0.06	0.25	-0.05	0.15	0.06	0.07	0.19	0.33
A13	0.05	0.07	0.22	0.01	-0.02	0.10	0.09	0.08	0.33	-0.01	0.15	0.06	1.00	-0.12	0.14	0.02	0.13	0.04	0.10	0.17
A14	0.11	0.17	-0.07	0.12	0.34	-0.13	0.22	0.13	-0.09	-0.07	-0.10	0.25	-0.12	1.00	-0.14	0.53	-0.05	-0.11	0.21	0.01
A15	0.01	-0.05	0.11	-0.05	-0.15	0.45	-0.01	0.05	0.11	0.38	0.00	-0.05	0.14	-0.14	1.00	-0.24	0.29	0.21	-0.06	0.16
A16	0.16	0.19	-0.10	0.15	0.41	-0.20	0.19	0.20	-0.08	-0.21	-0.15	0.15	0.02	0.53	-0.24	1.00	-0.15	-0.18	0.30	0.03
A17	0.01	0.05	0.20	0.00	-0.06	0.33	0.07	0.10	0.14	0.24	0.12	0.06	0.13	-0.05	0.29	-0.15	1.00	0.34	-0.07	0.23
A18	0.01	0.04	0.17	-0.07	-0.15	0.18	0.09	0.08	0.20	0.20	0.16	0.07	0.04	-0.11	0.21	-0.18	0.34	1.00	-0.16	0.31
A19	0.12	0.15	0.09	0.14	0.19	-0.05	0.28	0.26	-0.11	-0.14	-0.07	0.19	0.10	0.21	-0.06	0.30	-0.07	-0.16	1.00	-0.10
A20	0.08	0.10	0.19	0.08	0.13	0.20	0.31	0.05	0.10	0.13	0.18	0.33	0.17	0.01	0.16	0.03	0.23	0.31	-0.10	1.00

Note:
A1 to A20 denote items of the 20-item short-form of the Five-Facet Mindfulness Questionnaire.

Parallel Analysis of the FFMQ-V

Figure S1 shows the results of the parallel analysis of the FFMQ-V.

Figure S1: Parallel Analysis of the FFMQ-V

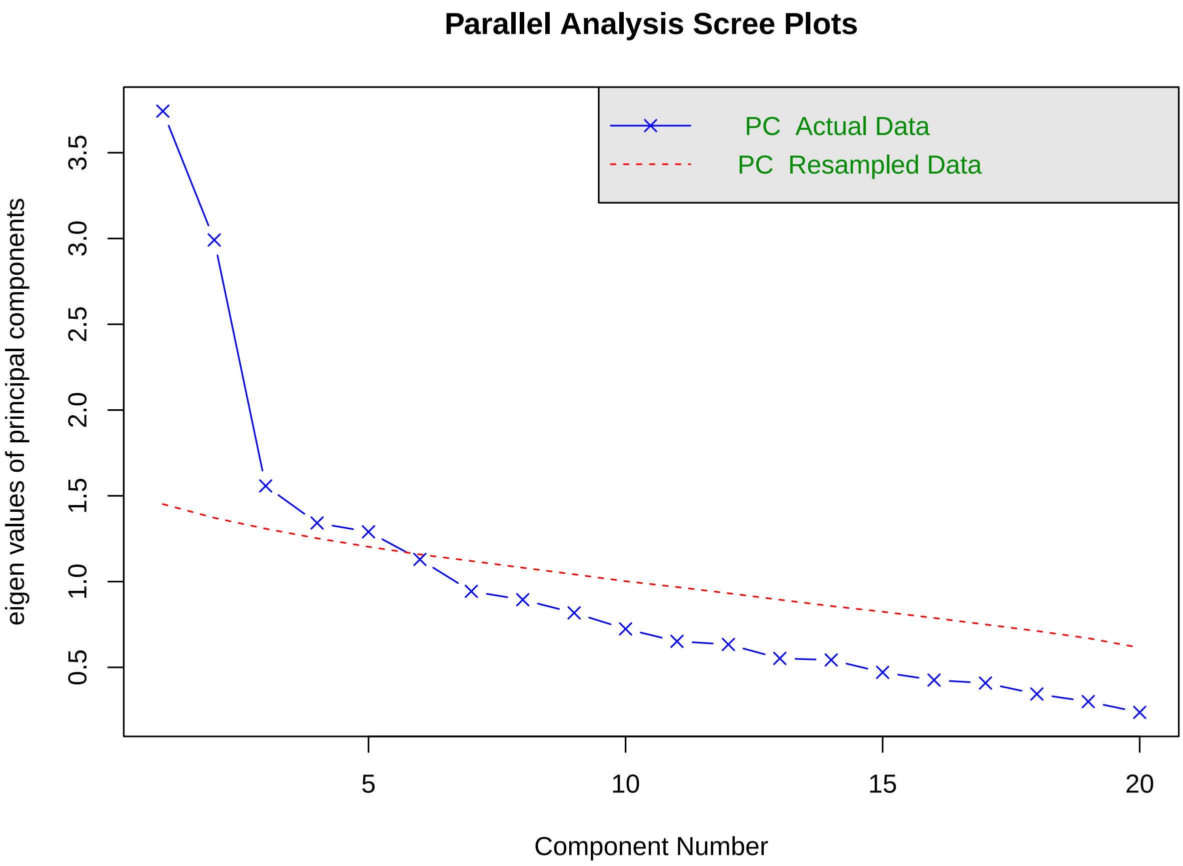

Note. PC denotes principle components and FA denotes factor analysis.

Score Differences of the FFMQ-V by Gender and Birth Place

For each item of the FFMQ-V, Table S2 reports the score mean and standard deviation by gender (i.e., female and male) and birth place (i.e., rural, urban, and mountainous areas)

Table S2: Average Score Differences by Gender and Birth Place of the FFMQ-V items

	Gender				Birth Place					
	Female		Male		Rural		Urban		Mountain	
	M	SD	M	SD	M	SD	M	SD	M	SD
A1	3.33	0.90	3.10	0.90	3.36	0.90	3.22	0.90	3.08	0.90
A2	3.01	0.97	2.89	0.87	3.07	0.95	2.94	0.95	2.71	0.94
A3	2.90	0.86	2.89	1.09	2.88	0.89	2.88	0.93	3.06	0.84
A4	3.41	0.95	3.26	1.04	3.46	0.95	3.33	1.02	3.06	0.86
A5	3.08	1.07	3.39	1.11	3.10	1.09	3.19	1.08	3.12	1.04
A6	3.50	1.22	3.50	1.07	3.50	1.19	3.54	1.19	3.44	1.27
A7	3.13	1.11	3.03	0.99	3.21	1.10	2.86	1.03	3.23	1.13
A8	2.80	0.98	2.61	0.89	2.82	1.02	2.69	0.94	2.69	0.78
A9	3.04	1.06	3.06	1.01	3.03	1.09	3.08	0.95	3.04	1.07
A10	3.28	1.14	3.19	1.13	3.17	1.16	3.39	1.10	3.48	1.03
A11	3.18	0.98	3.11	1.13	3.20	1.03	3.10	0.97	3.15	0.95
A12	2.91	0.99	2.55	0.94	2.90	0.99	2.71	0.98	2.96	0.99
A13	2.82	1.06	3.10	1.04	2.90	1.07	2.74	1.07	2.94	0.98
A14	3.28	1.06	3.11	1.07	3.25	1.07	3.22	1.10	3.33	1.00
A15	3.63	1.09	3.45	1.05	3.54	1.14	3.71	1.01	3.73	0.94
A16	3.21	1.11	3.06	0.99	3.17	1.11	3.15	1.07	3.40	1.03
A17	3.31	1.22	3.34	1.13	3.18	1.25	3.52	1.13	3.56	1.07
A18	3.51	1.05	3.77	0.89	3.53	1.06	3.63	1.00	3.52	0.97
A19	2.90	1.06	2.94	1.07	2.89	1.07	2.95	1.02	2.88	1.12
A20	3.01	1.09	3.15	0.99	2.94	1.08	3.18	1.05	3.19	1.08

Note:
M denotes the mean and SD denotes the standard deviation. A1 to A20 denote items of the 20-item short-form of the Five-Facet Mindfulness Questionnaire.

Study 2: A Confirmatory Factor Analysis in College Sample

In this section, we report additional Study 2 results that we performed in our analyses of the Vietnamese Five-Facet Mindfulness Questionnaire (FFMQ-V).

The Correlation Matrix of the FFMQ-V

Table S3 reports the polychoric correlations among FFMQ-V items.

Table S3: Pearson Correlations among FFMQ-V items in College Sample (N = 344)

	A1	A2	A3	A4	A5	A6	A7	A8	A9	A10	A11	A12	A13	A14	A15	A16	A17	A18	A19	A20
A1	1.00	0.72	0.11	0.59	0.18	0.00	0.22	0.47	0.04	-0.08	-0.14	0.25	0.13	0.24	-0.08	0.24	0.01	-0.06	0.23	0.10
A2	0.72	1.00	0.13	0.64	0.18	-0.01	0.26	0.49	0.04	-0.05	-0.14	0.32	0.05	0.23	-0.09	0.19	-0.05	-0.12	0.19	0.09
A3	0.11	0.13	1.00	0.19	0.00	0.11	0.04	0.05	0.40	0.13	0.17	0.04	0.14	0.19	0.12	-0.05	0.09	0.01	-0.03	0.13
A4	0.59	0.64	0.19	1.00	0.21	-0.02	0.28	0.49	0.03	0.04	-0.03	0.36	0.05	0.30	0.00	0.26	0.05	-0.19	0.23	0.15
A5	0.18	0.18	0.00	0.21	1.00	-0.10	0.28	0.28	0.00	-0.09	-0.08	0.17	0.08	0.44	-0.04	0.49	-0.07	-0.07	0.22	0.06
A6	0.00	-0.01	0.11	-0.02	-0.10	1.00	-0.15	0.00	0.09	0.41	0.11	0.02	0.07	0.02	0.34	-0.03	0.31	0.13	-0.04	0.12
A7	0.22	0.26	0.04	0.28	0.28	-0.15	1.00	0.35	-0.04	-0.18	-0.11	0.62	0.10	0.23	-0.05	0.34	-0.06	-0.01	0.17	0.31
A8	0.47	0.49	0.05	0.49	0.28	0.00	0.35	1.00	0.05	-0.03	-0.26	0.36	-0.03	0.31	0.03	0.27	0.02	-0.12	0.18	0.07
A9	0.04	0.04	0.40	0.03	0.00	0.09	-0.04	0.05	1.00	0.11	0.21	0.09	0.25	0.11	0.07	-0.05	0.22	0.22	-0.01	0.14
A10	-0.08	-0.05	0.13	0.04	-0.09	0.41	-0.18	-0.03	0.11	1.00	0.24	-0.12	0.06	-0.14	0.50	-0.10	0.30	0.17	-0.06	0.09
A11	-0.14	-0.14	0.17	-0.03	-0.08	0.11	-0.11	-0.26	0.21	0.24	1.00	-0.22	0.11	-0.11	0.29	-0.15	0.16	0.15	-0.20	0.09
A12	0.25	0.32	0.04	0.36	0.17	0.02	0.62	0.36	0.09	-0.12	-0.22	1.00	-0.04	0.30	-0.09	0.34	-0.01	0.04	0.22	0.34
A13	0.13	0.05	0.14	0.05	0.08	0.07	0.10	-0.03	0.25	0.06	0.11	-0.04	1.00	0.03	0.07	0.01	0.06	0.09	-0.02	0.13
A14	0.24	0.23	0.19	0.30	0.44	0.02	0.23	0.31	0.11	-0.14	-0.11	0.30	0.03	1.00	-0.11	0.54	-0.01	-0.08	0.23	0.13
A15	-0.08	-0.09	0.12	0.00	-0.04	0.34	-0.05	0.03	0.07	0.50	0.29	-0.09	0.07	-0.11	1.00	-0.11	0.47	0.31	-0.15	0.17
A16	0.24	0.19	-0.05	0.26	0.49	-0.03	0.34	0.27	-0.05	-0.10	-0.15	0.34	0.01	0.54	-0.11	1.00	-0.15	-0.17	0.28	-0.02
A17	0.01	-0.05	0.09	0.05	-0.07	0.31	-0.06	0.02	0.22	0.30	0.16	-0.01	0.06	-0.01	0.47	-0.15	1.00	0.38	-0.09	0.21
A18	-0.06	-0.12	0.01	-0.19	-0.07	0.13	-0.01	-0.12	0.22	0.17	0.15	0.04	0.09	-0.08	0.31	-0.17	0.38	1.00	-0.12	0.18
A19	0.23	0.19	-0.03	0.23	0.22	-0.04	0.17	0.18	-0.01	-0.06	-0.20	0.22	-0.02	0.23	-0.15	0.28	-0.09	-0.12	1.00	-0.01
A20	0.10	0.09	0.13	0.15	0.06	0.12	0.31	0.07	0.14	0.09	0.09	0.34	0.13	0.13	0.17	-0.02	0.21	0.18	-0.01	1.00

Note:

A1 to A20 denote items of the 20-item short-form of the Five-Facet Mindfulness Questionnaire.

Score Differences of the FFMQ-V by Gender and Birth Place

For each item of the FFMQ-V, Table S4 reports the score mean and standard deviation by gender (i.e., female and male) and birth place (i.e., rural, urban, and mountainous areas).

Table S4: Average Score Differences by Gender and Birth Place of the FFMQ-V items in College Sample (N = 344)

	Gender				Birth Place					
	Female		Male		Rural		Urban		Mountain	
	M	SD	M	SD	M	SD	M	SD	M	SD
A1	2.77	0.82	2.81	1.12	2.82	0.88	2.74	0.81	2.66	0.94
A2	3.03	0.85	3.16	0.95	3.06	0.90	3.03	0.79	2.98	0.85
A3	3.14	0.78	3.16	0.81	3.11	0.79	3.18	0.77	3.24	0.83
A4	2.66	0.87	2.77	1.02	2.67	0.90	2.76	0.92	2.51	0.78
A5	2.88	1.02	2.79	1.10	2.94	1.03	2.79	1.04	2.76	1.02
A6	3.51	0.99	3.53	0.93	3.53	1.01	3.50	0.93	3.41	0.97
A7	2.95	0.95	2.72	0.96	2.88	0.95	3.05	0.95	2.85	1.01
A8	3.13	0.87	2.72	1.10	3.05	0.93	3.07	0.91	3.20	0.84
A9	3.23	0.87	3.16	0.95	3.22	0.93	3.23	0.77	3.22	0.88
A10	3.20	0.96	3.07	0.99	3.12	0.97	3.29	0.95	3.24	0.97
A11	3.20	0.84	3.23	1.02	3.17	0.85	3.33	0.84	3.05	0.92
A12	3.07	0.96	3.12	0.96	3.00	0.99	3.24	0.86	3.07	0.98
A13	3.13	0.94	3.30	0.91	3.13	0.98	3.17	0.86	3.24	0.92
A14	2.68	0.99	2.81	1.05	2.67	0.99	2.76	0.99	2.66	1.09
A15	3.68	0.93	3.37	0.95	3.62	0.88	3.74	1.03	3.51	1.00
A16	2.76	0.95	2.65	0.90	2.74	0.94	2.79	0.96	2.68	0.96
A17	3.44	0.98	3.49	1.03	3.42	0.97	3.57	1.01	3.27	0.98
A18	3.64	0.90	3.47	0.98	3.56	0.92	3.71	0.92	3.66	0.88
A19	3.13	0.92	3.16	1.00	3.18	0.95	3.10	0.88	2.98	0.96
A20	3.20	0.91	3.35	0.92	3.17	0.94	3.31	0.89	3.24	0.83

Note:
M denotes the mean and SD denotes the standard deviation. A1 to A20 denote items of the 20-item short-form of the Five-Facet Mindfulness Questionnaire.

Study 3: A Confirmatory Factor Analysis in Community Sample

In this section, we report additional Study 3 results that we performed in our analyses of the Vietnamese Five-Facet Mindfulness Questionnaire (FFMQ-V).

The Correlation Matrix of the FFMQ-V

Table S5 reports the polychoric correlations among FFMQ-V items.

Table S5: Pearson Correlations among FFMQ-V items in Community Sample (N = 574)

	A1	A2	A3	A4	A5	A6	A7	A8	A9	A10	A11	A12	A13	A14	A15	A16	A17	A18	A19	A20
A1	1.00	0.65	-0.07	0.58	0.37	-0.17	0.36	0.44	0.00	-0.25	-0.21	0.29	0.08	0.19	-0.14	0.28	-0.12	-0.06	0.25	0.11
A2	0.65	1.00	-0.04	0.62	0.39	-0.17	0.40	0.52	0.02	-0.20	-0.21	0.36	0.06	0.12	-0.09	0.23	-0.10	-0.04	0.21	0.08
A3	-0.07	-0.04	1.00	-0.08	-0.17	0.18	-0.04	-0.08	0.30	0.21	0.27	-0.04	0.25	-0.04	0.12	-0.13	0.18	0.20	-0.13	0.20
A4	0.58	0.62	-0.08	1.00	0.39	-0.09	0.42	0.54	0.03	-0.20	-0.18	0.38	0.10	0.20	-0.08	0.26	-0.07	-0.12	0.21	0.12
A5	0.37	0.39	-0.17	0.39	1.00	-0.16	0.36	0.34	-0.13	-0.28	-0.24	0.27	0.05	0.27	-0.09	0.37	-0.13	-0.08	0.19	0.04
A6	-0.17	-0.17	0.18	-0.09	-0.16	1.00	-0.12	-0.06	0.20	0.42	0.22	-0.13	0.12	-0.13	0.39	-0.16	0.40	0.24	-0.30	0.17
A7	0.36	0.40	-0.04	0.42	0.36	-0.12	1.00	0.50	-0.05	-0.20	-0.24	0.51	0.09	0.29	-0.07	0.31	-0.11	-0.15	0.28	0.23
A8	0.44	0.52	-0.08	0.54	0.34	-0.06	0.50	1.00	0.02	-0.18	-0.16	0.56	0.08	0.13	0.00	0.22	-0.05	-0.01	0.22	0.17
A9	0.00	0.02	0.30	0.03	-0.13	0.20	-0.05	0.02	1.00	0.26	0.23	-0.09	0.29	-0.13	0.22	-0.18	0.24	0.31	-0.19	0.30
A10	-0.25	-0.20	0.21	-0.20	-0.28	0.42	-0.20	-0.18	0.26	1.00	0.35	-0.23	0.10	-0.17	0.34	-0.28	0.33	0.17	-0.25	0.12
A11	-0.21	-0.21	0.27	-0.18	-0.24	0.22	-0.24	-0.16	0.23	0.35	1.00	-0.26	0.16	-0.21	0.20	-0.27	0.18	0.17	-0.19	0.13
A12	0.29	0.36	-0.04	0.38	0.27	-0.13	0.51	0.56	-0.09	-0.23	-0.26	1.00	-0.05	0.21	-0.04	0.34	-0.10	-0.05	0.22	0.17
A13	0.08	0.06	0.25	0.10	0.05	0.12	0.09	0.08	0.29	0.10	0.16	-0.05	1.00	-0.21	0.18	-0.13	0.18	0.25	-0.07	0.32
A14	0.19	0.12	-0.04	0.20	0.27	-0.13	0.29	0.13	-0.13	-0.17	-0.21	0.21	-0.21	1.00	-0.24	0.43	-0.15	-0.23	0.31	-0.13
A15	-0.14	-0.09	0.12	-0.08	-0.09	0.39	-0.07	0.00	0.22	0.34	0.20	-0.04	0.18	-0.24	1.00	-0.24	0.34	0.29	-0.23	0.27
A16	0.28	0.23	-0.13	0.26	0.37	-0.16	0.31	0.22	-0.18	-0.28	-0.27	0.34	-0.13	0.43	-0.24	1.00	-0.21	-0.29	0.33	-0.13
A17	-0.12	-0.10	0.18	-0.07	-0.13	0.40	-0.11	-0.05	0.24	0.33	0.18	-0.10	0.18	-0.15	0.34	-0.21	1.00	0.38	-0.23	0.22
A18	-0.06	-0.04	0.20	-0.12	-0.08	0.24	-0.15	-0.01	0.31	0.17	0.17	-0.05	0.25	-0.23	0.29	-0.29	0.38	1.00	-0.34	0.32
A19	0.25	0.21	-0.13	0.21	0.19	-0.30	0.28	0.22	-0.19	-0.25	-0.19	0.22	-0.07	0.31	-0.23	0.33	-0.23	-0.34	1.00	-0.15
A20	0.11	0.08	0.20	0.12	0.04	0.17	0.23	0.17	0.30	0.12	0.13	0.17	0.32	-0.13	0.27	-0.13	0.22	0.32	-0.15	1.00

Note:
A1 to A20 denote items of the 20-item short-form of the Five-Facet Mindfulness Questionnaire.

Score Differences of the FFMQ-V by Gender, Birth Place, and Occupation

For each item of the FFMQ-V, Table S6 reports the score mean and standard deviation by gender (i.e., female and male), birth place (i.e., rural, urban, and mountainous areas), and occupation (i.e., students, labor workers, and intellectuals).

Table S6: Average Score Differences by Gender, Birth Place, and Occupation of the FFMQ-V items in Community Sample (N = 574)

	Gender				Birth Place						Occupation					
	Female		Male		Rural		Urban		Mountain		Students		Labors		Intellectuals	
	M	SD	M	SD	M	SD	M	SD	M	SD	M	SD	M	SD	M	SD
A1	3.03	0.96	3.22	1.06	3.00	1.04	3.18	0.94	3.29	1.27	2.91	1.02	3.55	0.94	3.23	0.81
A2	3.41	0.98	3.51	1.03	3.31	1.01	3.56	0.96	3.62	1.07	3.25	0.97	3.95	0.92	3.41	0.73
A3	2.96	1.06	2.88	1.06	2.93	1.07	2.94	1.05	2.82	1.11	2.87	1.02	3.02	1.20	2.82	0.73
A4	3.02	1.10	3.16	1.11	3.01	1.08	3.11	1.09	3.29	1.34	2.85	1.10	3.53	1.05	3.23	0.87
A5	3.05	1.24	3.21	1.22	3.08	1.25	3.17	1.21	2.97	1.34	2.98	1.23	3.38	1.33	3.00	0.98
A6	3.48	1.27	3.17	1.27	3.33	1.24	3.37	1.31	3.29	1.31	3.44	1.25	3.22	1.42	3.50	1.14
A7	3.03	1.28	3.06	1.29	3.05	1.26	3.02	1.30	3.24	1.33	2.81	1.29	3.36	1.32	3.14	1.21
A8	3.49	1.08	3.49	1.15	3.40	1.16	3.56	1.06	3.56	1.11	3.30	1.13	3.97	0.97	3.50	1.14
A9	3.23	1.18	3.15	1.27	3.07	1.17	3.27	1.27	3.41	1.10	3.19	1.17	3.41	1.35	2.91	1.06
A10	3.11	1.19	2.89	1.19	3.08	1.19	2.94	1.20	3.12	1.20	3.08	1.21	2.91	1.21	3.41	1.26
A11	3.10	1.16	3.03	1.13	3.06	1.11	3.07	1.19	3.18	1.09	3.14	1.13	2.93	1.25	3.09	0.97
A12	3.21	1.19	3.22	1.17	3.23	1.15	3.19	1.21	3.26	1.14	3.06	1.18	3.58	1.25	2.91	0.97
A13	2.97	1.18	3.14	1.20	2.93	1.13	3.16	1.21	2.94	1.32	2.92	1.18	3.27	1.25	2.86	1.17
A14	2.73	1.17	2.80	1.12	2.77	1.12	2.74	1.20	2.85	1.05	2.76	1.15	2.60	1.25	2.86	0.99
A15	3.46	1.12	3.29	1.19	3.41	1.14	3.38	1.15	3.26	1.33	3.38	1.16	3.38	1.25	3.55	1.01
A16	2.78	1.14	2.87	1.19	2.89	1.20	2.78	1.11	2.50	1.21	2.74	1.17	2.98	1.30	2.45	0.91
A17	3.25	1.19	3.07	1.25	3.08	1.20	3.25	1.23	3.12	1.30	3.18	1.18	3.15	1.41	3.41	1.05
A18	3.63	1.02	3.49	1.14	3.47	1.10	3.62	1.05	3.88	0.95	3.54	1.05	3.77	1.12	3.68	0.95
A19	2.99	1.22	3.15	1.16	3.06	1.20	3.06	1.20	3.03	1.14	3.02	1.21	3.12	1.30	3.18	0.85
A20	3.27	1.00	3.25	1.08	3.19	0.95	3.33	1.09	3.24	1.10	3.14	0.98	3.52	1.19	3.32	1.04

Note:
M denotes the mean and SD denotes the standard deviation. A1 to A20 denote items of the 20-item short-form of the Five-Facet Mindfulness Questionnaire.
